# Roles of M1 Macrophages and Their Extracellular Vesicles in Cancer Therapy

**DOI:** 10.3390/cells13171428

**Published:** 2024-08-26

**Authors:** Wenli Zhou, Fengtang Yang, Xiuzhen Zhang

**Affiliations:** School of Life Sciences and Medicine, Shandong University of Technology, Zibo 255049, Chinafengtangyang@163.com (F.Y.)

**Keywords:** M1 macrophage, M2 macrophage, cancer, extracellular vesicles, exosome

## Abstract

Tumor-associated macrophages (TAMs) are inflammatory cells that are important components of the tumor microenvironment. TAMs are functionally heterogeneous and divided into two main subpopulations with distinct and opposite functions: M1 and M2 macrophages. The secretory function of TAMs is essential for combating infections, regulating immune responses, and promoting tissue repair. Extracellular vesicles (EVs) are nanovesicles that are secreted by cells. They play a crucial role in mediating intercellular information transfer between cells. EVs can be secreted by almost all types of cells, and they contain proteins, microRNAs, mRNAs, and even long non-coding RNAs (lncRNAs) that have been retained from the parental cell through the process of biogenesis. EVs can influence the function and behavior of target cells by delivering their contents, thus reflecting, to some extent, the characteristics of their parental cells. Here, we provide an overview of the role of M1 macrophages and their EVs in cancer therapy by exploring the impact of M1 macrophage-derived EVs (M1-EVs) on tumors by transferring small microRNAs. Additionally, we discuss the potential of M1-EVs as drug carriers and the possibility of reprogramming M2 macrophages into M1 macrophages for disease treatment. We propose that M1-EVs play a crucial role in cancer therapy by transferring microRNAs and loading them with drugs. Reprogramming M2 macrophages into M1 macrophages holds great promise in the treatment of cancers.

## 1. Introduction

**Figure 1:**
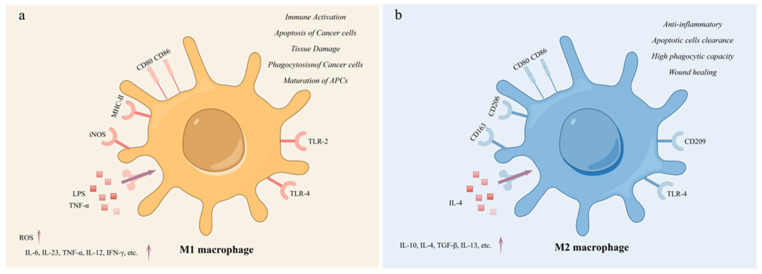
The characteristics of M1 and M2 macrophages. (**a**) M1 Macrophage; (**b**) M2 macrophage. M1 macrophages were mainly activated by LPS and TNF-α and secreted pro-inflammatory factors. M1 macrophages are involved in immune activation, promoting apoptosis and phagocytosis of cancer cells. M2 macrophages were mainly activated by IL-4 and mainly secreted anti-inflammatory factors, with high expression of CD206 and enhanced endocytosis. M2 macrophages have anti-inflammatory activity and participate in wound damage repair, phagocytosis, and clearance of apoptotic cells.

Macrophages are highly heterogeneous and plastic immune cells that play an important role in clearing pathogens and cellular waste in the body. Macrophages can be classified into M0, M1, and M2 types based on their phenotypic and functional characteristics [1]. M0 macrophages are inactive macrophages, usually in a quiescent state. M0 macrophages perform basic immune surveillance and maintain tissue homeostasis in tissues. They can remove pathogens and apoptotic cells through phagocytosis, but their activity is lower when not stimulated [2]. Tumor-associated macrophages (TAMs) are a crucial component of the tumor microenvironment (TME). They have a significant impact on tumor growth, immune regulation, and chemotherapy resistance [3]. In general, TAMs refer to all macrophages that function in the tumor microenvironment and exert different pro- or antitumor effects [4]. This polarization occurs due to factors such as the environment in which they reside [5]. TAMs polarize into two phenotypes: M1 macrophages and M2 macrophages [6]. M1 and M2 macrophages represent the two extremes of the macrophage functional spectrum [7]. M1 macrophages are usually induced by stimuli such as tumor necrosis factor (TNF)-α and lipopolysaccharide (LPS) [8]. M1 macrophages produce high levels of reactive oxygen species (ROS) in their activated state, which are believed to kill tumor cells and play an important role in antitumor activity and immune enhancement [9]. These cells secrete various pro-inflammatory cytokines, such as IL-12, TNF-α, IL-6, IL-23, and IFN-γ, and exhibit high levels of inducible nitric oxide synthase (iNOS) production (Figure 1a) [10]. M1 macrophages have strong phagocytic and bactericidal abilities and participate in stimulating adaptive immune responses, particularly the activation of Th1 cells [11]. In contrast, M2 macrophages are usually induced by stimuli such as IL-4. M2 macrophages exhibit immunosuppression and promote tissue repair and tumor development. These cells secrete anti-inflammatory cytokines, such as IL-10, IL-13, TGF-β, and IL-4, and they express specific markers such as CD206 (Figure 1b). M2 also inhibits Th1 cell activation and promotes Th2 cell response [12]. Both M1 and M2 macrophages are present throughout all stages of the tumor. However, M1 macrophages are more prevalent in the early stages, while M2 macrophages are more prevalent in the middle and late stages [13]. As the tumor progresses, M1 macrophages are increasingly polarized towards M2 macrophages. The increase in M2 macrophage count is associated with poor prognosis [14].

Under physiological and pathological conditions, macrophages can change M1 and M2 states by receiving different stimuli according to their environment [15]. This plasticity allows macrophages to perform multiple functions in different situations to maintain organismal homeostasis [16]. Macrophage polarization plays a key role in the occurrence and progression of multiple diseases; therefore, regulating the polarization status of macrophages is important in therapy [17]. In the tumor microenvironment, the polarization status of macrophages directly affects tumor growth and spread. M2 macrophages usually promote tumor growth and immune escape. Therefore, transforming macrophages from M2 to M1 by drugs or other means can promote the antitumor immune response and improve therapeutic efficacy [18]. In chronic inflammatory diseases, excessive inflammatory responses can lead to tissue damage. By promoting macrophage polarization towards M2, inflammation can be effectively attenuated, and tissue repair and recovery can be promoted [19]. In wound repair and tissue engineering, regulating the polarization of macrophages can improve wound healing and tissue regeneration. M2 macrophages play an important role in this process by promoting their polarization, which can accelerate wound healing and tissue reconstruction [20]. In certain infectious diseases, the polarization state of macrophages determines their ability to clear pathogens. By regulating the polarization of macrophages, their phagocytic and bactericidal abilities can be enhanced, increasing the body’s resistance to infections [21]. In conclusion, macrophage polarization has a wide range of applications.

## 2. M1 Macrophage-Derived Extracellular Vesicles

### 2.1. Tumor Cells and the Tumor Microenvironment

Tumors are a significant health concern. According to GLOBOCAN 2022, there will be close to 20 million new tumor cases, with 9.7 million resulting in death. The global cancer burden is projected to continue to increase in the coming decades. These data reflect the severe global burden of cancer and highlight the importance of prevention, early diagnosis, and treatment [22]. Cancer cells possess a robust capacity for proliferation, migration, and invasion, which is challenging to regulate [23]. In addition to the properties of the cancer cells themselves, there is increasing evidence that TME also plays a crucial role in the development, progression, metastasis, and drug resistance of tumors [24]. The TME includes surrounding immune cells, blood vessels, fibroblasts, inflammatory cells, signaling molecules, and extracellular matrix [25], which provide favorable conditions for the development of tumor cells. The TME plays an active role in driving cancer progression rather than simply being a passive bystander [26]. In the late 19th century, Stephen Paget proposed the “seed and soil” hypothesis, which is now the basis for the concept of tumor microenvironment. This hypothesis considers tumor cells as “seeds” and the tumor microenvironment as the soil. The survival and growth of the seeds are closely related to the fertility of the soil, and the seeds and the soil interact and influence each other [27]. Thus, the microenvironment of a tumor could have a significant impact on both the response to treatment and clinical outcomes [28].

### 2.2. Cells Secrete Extracellular Vesicles to Transmit Biological Signals and Regulate the Surrounding Microenvironment

Extracellular vesicles (EVs) can be classified according to the cellular pathways involved in their biogenesis [29]. Currently, the most widely studied subtype of EVs are exosomes with a diameter of 30–150 nm [30]. The concept of exosome was first introduced in 1983 by a team of researchers studying sheep erythrocytes. They found these vesicles in the culture supernatant of sheep erythrocytes and concluded that they were small vesicles of cells with membrane structures [31]. In 1987, Johnstone named them “exosomes” [32]. Like cell membranes, they have a phospholipid bilayer that appears as a saucer under the electron microscope [33]. The exosomes exhibit low immunogenicity and high biocompatibility as they are produced endogenously by the cells themselves [34]. Initially, exosomes were considered intracellular “junk” and received little research attention until recent studies showed that exosomes contain various RNAs, ncRNAs, and proteins that participate in cell communication, as witnessed by the huge increase in the number of papers studying exosomes [35]. Exosomes promote intercellular communication by transporting RNA and proteins to neighboring cells and distant organs [36]. Most cells are able to actively secrete exosomes into bodily fluids, such as blood, milk, and amniotic fluid [37]. In addition, exosomes are closely related to tumor development and can serve as liquid biopsies and non-invasive biomarkers [38]. The current isolation techniques often fail to distinguish between various vesicle types. The International Society for Extracellular Vesicles (ISEV) community recommends using “EVs” for vesicles isolated from cell culture supernatants or body fluids [39].

EVs participate in exchanging information between tumor cells and TAMs [40]. EVs are paracrine substances that contain mRNA, microRNA, proteins, and other biomolecules retained by the parental cell [41]. Tumor cells can transfer oncogenes by secreting EVs. They can also change the tumor environment to promote tumor proliferation and development. Additionally, they can weaken the immune response by transmitting signaling factors that inhibit the tumor immune response [42]. Tumor-secreted EVs may contribute to immune escape and immune tolerance in tumors [43]. Meanwhile, numerous studies have demonstrated that macrophages derived EVs are involved in regulating inflammatory processes and are involved in the development of various diseases, including cancer, atherosclerosis, diabetes, and heart failure [44]. When macrophages are polarized, EVs secreted by M1 macrophages can act as immune enhancers to produce cancer vaccines. This induces the generation of toxic cells to combat cancer cells [45].

### 2.3. EVs Signal by Transferring microRNAs

MicroRNAs are a class of endogenous small RNAs, measuring approximately 20–24 nucleotides in length [46]. They are derived from transcripts that form a unique hairpin structure called pre-microRNAs [47]. MicroRNAs play key roles in the post-transcriptional regulation of gene expression. Target gene silencing results from the complementary sequence pairing between microRNAs and the 3′UTR of target mRNA transcripts [48]. They participate in regulating the expression of up to 30% of mammalian protein-coding genes [49].

Research has shown that many non-coding RNAs present in EVs are microRNAs, and microRNAs could constitute up to 50% of non-coding RNAs, depending on cellular origin [50,51]. In recent years, EV microRNAs have attracted great attention because the loading of microRNAs into EVs is not a random process [52]. Many studies have shown that EVs function by delivering microRNAs [53]. MicroRNAs have great potential for diagnostic and therapeutic applications.

### 2.4. M1-EVs Show Promise in Cancer Models through microRNA Transfer

**Figure 2:**
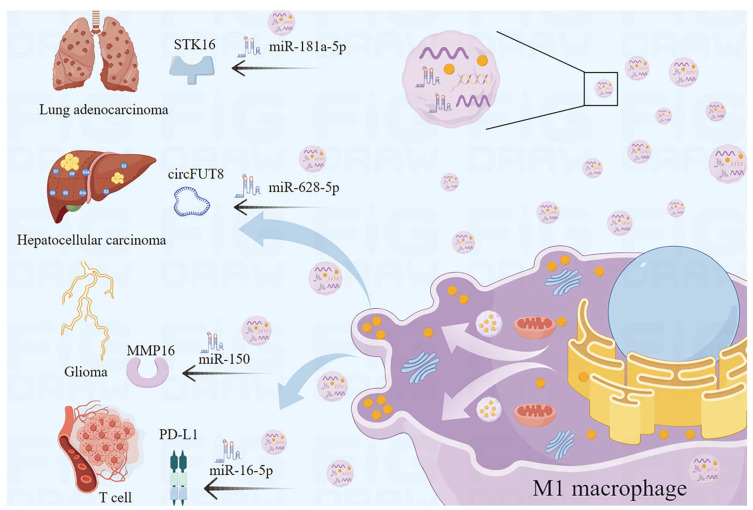
M1-EVs function by microRNA transfer. M1-EVs delay and inhibit the progression of multiple cancers through microRNA transfer.

EVs secreted by M1 macrophages can transfer microRNA and affect disease progression in various ways [54,55]. M1-EVs can inhibit tumor development by regulating enzymes related to cancer cell proliferation, migration, invasion, and apoptosis through microRNA transfer. Matrix metalloproteinase-16 (MMP-16) is a membrane-bound metalloproteinase that is associated with the proliferation, invasion, and migration of cancer cells [56]. Yan et al. demonstrated that the EVs miR-150 from M1 macrophages enter glioma cells, bind to MMP-16, downregulate its expression, and inhibit glioma progression [57]. Serine/threonine protein kinase 16 (STK16) plays a critical role in regulating tumor cell proliferation, apoptosis, and prognosis [58]. Wang et al. demonstrated that miR-181a-5p, present in M1-EVs, inhibited cell proliferation and promoted apoptosis by targeting STK16 [59]. In addition, M1-EVs can hinder the modifying effects of tumor-associated molecules through microRNA transfer, therefore hindering disease progression. Wang et al. demonstrated that EVs miR-628-5p, derived from M1 macrophages, inhibited the m6A modification of circFUT8, therefore suppressing the development of hepatocellular carcinoma [60]. Furthermore, studies have shown that M1-EVs could affect signaling pathways associated with cell proliferation, differentiation, apoptosis, and migration through microRNA transfer, ultimately inhibiting cancer progression. Li et al. found that M1-EVs carrying miR-16-5p activated T-cell immune responses by reducing PD-L1, which inhibits the process of gastric cancer [54] (Figure 2). Increasing attention has been paid to the differences between M1-EVs and M2 macrophage-derived EVs to explore anti-cancer molecules that play key roles in EVs. In conclusion, more and more studies indicate that M1-EVs have therapeutic effects in cancer through microRNA transfer.

## 3. M1-EVs Loaded with Drugs Act Synergistically to Fight Cancer

### 3.1. Advantages of M1-EVs as Drug Carriers

**Figure 3:**
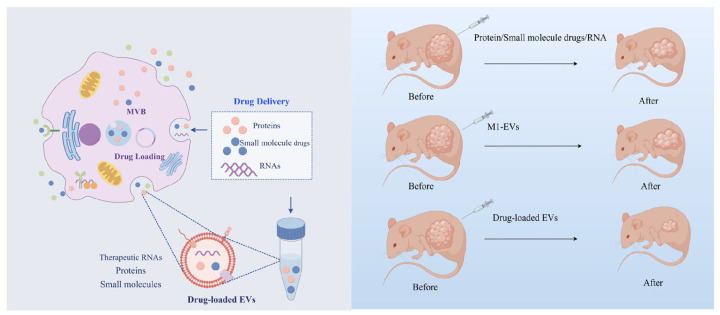
M1-EVs have shown better therapeutic efficacy as drug carriers in disease treatment. EVs produced by M1 macrophages can be used as novel drug carriers to encapsulate proteins, therapeutic RNAs, and small-molecule drugs to reach the lesion site. Compared with EVs alone or these drugs alone, EV-loaded drugs have better synergistic therapeutic effects.

Cells can secrete and absorb EVs, making them practical drug carriers [61]. EVs have unique advantages as drug carriers [62]. First, as EVs are secreted by cells, they can avoid being degraded by phagocytosis as foreign substances after entering the organism and will not produce immune rejection [63]. Second, the ability of EVs to survive under acidic and digestive enzyme conditions opens the possibility of oral drug formulations [64]. EVs from various cell sources can target different receptor cells, reducing the toxic side effects of traditional drugs and improving drug efficiency [65]. In addition, due to their nanoscale size, EVs can cross the blood-brain barrier, providing potential treatment options for neurological diseases [66]. EVs possess the properties required for use as drug carriers. Since 2010, attempts have been made to treat inflammatory diseases using EVs delivery of curcumin. It has been found that the anti-inflammatory activity of curcumin encapsulated in vesicles is more than three times stronger than that of delivered alone [67]. EVs can deliver three types of drugs: small-molecule drugs, such as anti-inflammatory and anti-cancer drugs, nucleic acid drugs for gene therapy, and large protein drugs [68] (Figure 3).

M1-EVs are a good choice for cancer drug carriers. First, M1-EVs are enriched with pro-inflammatory factors and immune-activating molecules, such as TNF-α, IL-12, and iNOS, which enhance antitumor immune responses and activate the immune system. Therefore, they have great potential for application in cancer immunotherapy [69]. Second, M1 macrophages have significant tumor suppressor and tumor targeting abilities. M1-EVs efficiently take up antigens and present them to helper T-cells via MHC-II molecules, triggering a strong immune response [45]. Finally, the resistance of tumor cells to conventional chemotherapeutic drugs can be reduced using M1-EVs as drug carriers. This is because EVs can enter cells through different pathways, bypassing traditional drug resistance mechanisms [70]. Therefore, M1-EVs are a suitable option as drug carriers. M1-EVs can be used to treat various types of tumors by delivering endogenous or exogenous microRNAs and proteins.

### 3.2. M1-EVs Loaded with Drugs to Achieve Better Therapeutic Effects

Pancreatic cancer is a common malignant tumor of the digestive system [71]. Pancreatic cancer is widely recognized as one of the deadliest cancers due to its highly malignant nature, difficulty in early diagnosis, and extremely poor prognosis [72]. Gemcitabine (GEM) is currently used as a first-line chemotherapeutic agent. However, its therapeutic efficacy is limited due to issues such as resistance and targeting [73]. The study demonstrated that the utilization of M1-EVs as a drug carrier, combined with deferasirox (DFX), enhanced the chemosensitivity of GEM by reducing the expression of ribonucleotide reductase regulatory subunit M2 (RRM2), which is suppressed by iron depletion [74]. Similarly, when bladder cancer cells were treated with gemcitabine loaded with M1-EVs using ultrasound technology, the results showed that M1-EVs-GEM significantly inhibited tumor growth and destroyed tumor cells. This treatment was found to be superior to GEM alone. It also raises the levels of inflammatory cytokines in the tissues [75]. In both in vivo and ex vivo experiments, it was demonstrated that loading cisplatin into M1-EVs has the potential to inhibit tumor growth. In addition, M1-EVs can be used as a carrier to encapsulate DDP and enhance its anti-lung cancer effects [76]. The DTX-M1-EVs drug-delivery system, consisting of M1-EVs as a carrier loaded with docetaxel (DTX), not only showed an effective inhibitory effect on pancreatic cancer but also induced the polarization of M0 macrophages to the M1 phenotype. This suggests that DTX-M1-EVs can achieve long-term effective M1 activation in immunosuppressive tumor microenvironments and can achieve significant antitumor therapeutic effects by combining chemotherapy and immunotherapy [69]. The different cargoes delivered by M1-EVs for various tumor treatments are shown in Table 1.

### 3.3. Future Perspectives of M1-EVs as Drug Carriers

Although EVs have many advantages as drug carriers, their clinical application still has a long way to go. The large-scale production of EVs remains a technical challenge, which greatly limits the in vivo applications of EVs [79]. Only a limited number of cell types can secrete enough EVs for clinical transformation [80]. To produce clinical-grade doses of EVs, a huge number of cells are typically cultured for several days [81]. EVs purification is then performed and nucleic acids, proteins, small-molecule drugs, and other substances are encapsulated into EVs [82]. To realize the potential of M1-EVs as drug carriers, many difficult tasks await to be completed, such as optimizing the encapsulation of specific drugs [83], enhancing the ability of cells to secrete EVs [84], rapidly and effectively obtaining huge quantities of high-purity EVs [85], and inducing the rapid release of the contents of EVs into the cytoplasm [86].

## 4. Reprogramming of M2 Macrophages into M1 Macrophages to Fight Cancer

### 4.1. Reprogramming M2 to M1 Macrophages: A New Strategy for Cancer Treatment

**Figure 4:**
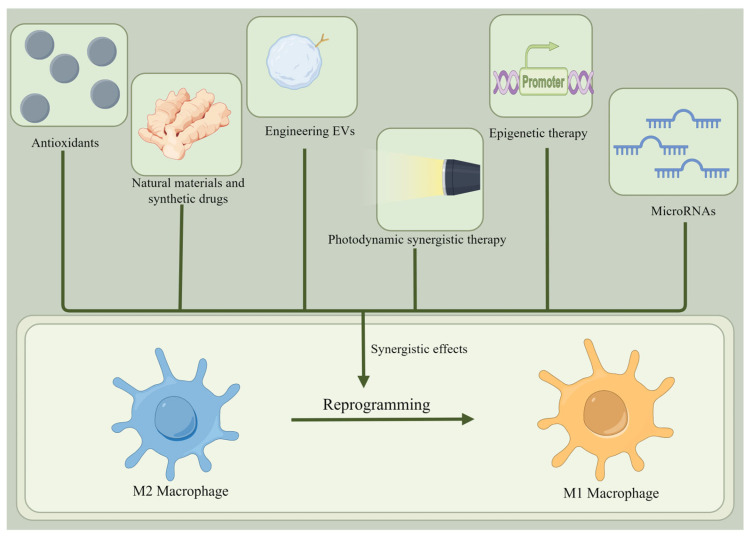
Reprogramming of M2 macrophages into M1 macrophages. There are many ways to reprogram M2 macrophages into M1 macrophages, such as using antioxidants, natural materials, synthetic drugs, engineered EVs, photodynamic synthetic therapies, epigenetic therapies, and microRNAs.

It is worth noting that M2 macrophages are more plastic and can readily repolarize to an inflammatory M1 state [87]. Thus, it is feasible to use a therapeutic strategy to re-differentiate the pro-tumor M2 phenotype into an antitumor M1 phenotype, inhibit TAMs, and slow tumor progression [88]. Theoretically, reprogramming M2 macrophages into M1 macrophages involves inhibiting the markers and cytokines of M2 macrophages and increasing those of M1 macrophages [89]. There are several ways to reprogram M2 macrophages into M1 macrophages (Figure 4 and Table 2).

### 4.2. Antioxidants

There is a complex relationship between ROS levels and cancer [90]. A certain level of ROS is crucial for maintaining normal cellular physiological activities. Generally, moderate levels of ROS can cause cell damage, therefore promoting the occurrence of cancer. Excessive levels of ROS can lead to cancer cell death, demonstrating anti-cancer effects [92].

Under normal conditions, there is a dynamic balance between ROS production and clearance, but this balance is often disturbed in cancer [93]. In cancer development, tumor cells may produce more ROS, which can promote tumor growth and progression [94].

Several studies have reported that different lipid peroxide scavengers can reprogram macrophages from M2 to M1 macrophages [95]. M1 and M2 macrophages have different pro-oxidase and antioxidant profiles, so their sensitivity to ROS regulation is also different [96]. ROS levels in M2 macrophages were significantly lower than those in M1 macrophages [97]. M2 macrophages have increased ROS metabolism, which may provide an advantage in the oxidative tumor microenvironment [98]. In addition, the oxidized tumor microenvironment promotes the generation of M2 macrophages [99]. Therefore, the use of antioxidants can reduce extracellular oxidative stress, leading to a decrease in M2 macrophage markers and an increase in M1 macrophage markers, ultimately successfully reprogramming M2 macrophages into M1 macrophages [95]. At present, the method of transforming M2 macrophages into M1 phenotype using antioxidants has not been widely studied. Therefore, the application of antioxidants requires a certain degree of caution and must be determined according to the specific background and objectives of the study [100].

### 4.3. Photodynamic Synergistic Therapy

Photodynamic therapy (PDT) utilizes photosensitizers to produce cytotoxic ROS, which helps destroy cancer cells and other pathological tissues [101]. Studies have revealed that the ROS generated during PDT can facilitate the reprogramming of macrophages towards the M1 phenotype [102]. Consequently, PDT represents a promising strategy for cancer treatment [103].

For instance, Guang et al. designed three photosensitizers with varying ROS generation efficiencies and observed that type I photosensitizers, through the production of free radicals, can reprogram M2 macrophages into the M1 phenotype. This reprogramming effectively inhibits tumor growth in vivo [102]. It is important to note that ROS can be classified into two categories. Type I ROS, formed via electron transfer by tri-linear photosensitizers, primarily comprises superoxide anion radicals, hydrogen peroxide, and hydroxyl radicals. In contrast, type II ROS is predominantly singlet oxygen generated through energy transfer from oxygen [104].

The extracellular ROS generated by type I photosensitizers is chiefly responsible for reprogramming macrophages from a pro-tumor (M2) to an antitumor (M1) state [105]. This strategy proves highly effective in overcoming immune suppression within the tumor microenvironment, therefore offering significant potential for improving cancer treatment outcomes [106].

### 4.4. Epigenetic Therapy

The core of epigenetics is the covalent modification of histones and nucleic acids, which cooperatively regulate chromatin structure and gene expression [107]. Dysregulation of the epigenome promotes cancer development and progression through aberrant transcriptional programmers [108]. Epigenetic therapies have received significant attention in recent years due to their improved precision and therapeutic efficacy compared to traditional drugs [109]. DNA methylation modulators and bromodomain extra-terminal (BET) inhibitors are two of the most popular research areas in the field of epigenetics [110]. Genetic mechanisms play a pivotal role in regulating and transmitting signals during macrophage polarization and reprogramming [111]. Epigenetic therapies can be used to reprogram M2 macrophages, as demonstrated [112,113,114]. Sri et al. increased the expression of miR-7083-5p in M2 macrophages by inhibiting DNA methylation and histone deacetylation. This reprogrammed M2 macrophages into M1 macrophages, resulting in inhibited tumor growth and increased sensitivity of tumor cells to the drug [112]. The investigators provide evidence that immunomodulatory drug epigenetically interferes with IFN regulatory factors 4 and 5 through degradation of IKAROS family zinc finger 1, which in turn alters the balance of M1/M2 polarization [113]. Li et al. showed that LARRPM exerts epigenetic regulation on LINC00240 and CSF1 to limit lung adenocarcinoma progression and M2 macrophage polarization [114]. Epigenetic therapies enable reprogramming of M2 macrophages to M1 macrophages, providing an excellent opportunity for therapeutic intervention.

### 4.5. Natural Materials and Synthetic Drugs

In addition, studies have demonstrated that several drugs can convert M2 macrophages into M1 macrophages [115]. This includes natural biological herbs and synthetic drugs. In cancer therapy research, targeting molecules and signaling pathways that participate in macrophage polarization have been widely used to reprogram M2 macrophages [116]. Yao et al. extracted 6-gingerol from ginger, which acted as an arginase inhibitor. This effectively prevented lung cancer by reprogramming tumor-promoting M2 macrophages into antitumor M1 macrophages [117]. Paclitaxel, a natural broad-spectrum anti-cancer drug, reprogrammed M2-polarized macrophages to an M1-like phenotype in a TLR4-dependent manner, resulting in tumor growth inhibition [118]. Zoledronic acid (ZA) is a bisphosphonate that strongly inhibits bone resorption and, potentially, bone formation. When ZA acts on macrophages, TLR-4 expression is elevated, leading to increased M1 macrophage polarization in vitro and in vivo [119]. Chlorogenic acid (5-caffeoylquinic acid, CHA) is a phenolic compound with a low molecular weight that has shown antitumor effects in various malignancies [120]. According to Xue et al., Chlorogenic acid mediates the JAK-STAT1 and NF-κB pathways to promote polarization of mouse bone marrow macrophages towards the M1 phenotype [121]. In addition, the use of agonist antibodies targeting CD40 is a promising approach for cancer immunotherapy [122]. CD40 agonists were found to alter the pancreatic cancer microenvironment by shifting macrophage phenotype towards M1 and inhibiting human pancreatic cancer in organ section culture [123]. In 2016, a study using a mouse tumor model demonstrated that histidine-rich glycoprotein (HRG), a plasma protein derived from the liver, facilitates the transition from alternatively activated (M2) to pro-inflammatory (M1) macrophages. This transition helps to limit tumor growth and metastasis [124]. Therefore, HRG is one of the effective drugs to induce TAM differentiation. Gemcitabine, commonly used in pancreatic cancer chemotherapy [125], and fluorouracil in bowel cancer chemotherapy, as well as classical platinum-containing two-agent chemotherapy [126], can polarize TAMs from the M2 to the M1 phenotype, thus promoting antitumor immunity. Lurbinectedin, an FDA-approved chemotherapy drug for lung cancer, is capable of inducing apoptosis in TAMs directly [127]. Several studies are currently investigating methods to regulate macrophage polarization, including the use of small-molecule compounds and antibody drugs. Research targeting macrophage polarization is gaining ground and promises to lead to the development of effective drugs for the treatment of diseases in the future. Therefore, this is a promising area of research that deserves continued attention. Table 3 summarizes some molecules and their targets that participate in macrophage reprogramming.

### 4.6. Engineering EVs

In addition to chemically synthesized drugs, engineered EVs are also effective tools for reprogramming M2 macrophages into M1 macrophages. EVs produced by M1 macrophages, including those carrying OX40L and IL4RPep-1 [133], have been shown to successfully reprogram M2 macrophages into M1 macrophages, enhancing M1 macrophage-mediated innate immunity. This provides a promising new approach for cancer treatment [137]. It is possible that engineered EVs could be used to influence epigenetic regulation and gene expression in macrophages, therefore facilitating the conversion of M2 macrophages to M1 macrophages [138]. This strategy utilizes EVs as natural messengers to encapsulate and deliver therapeutic molecules inside target cells [139]. Overall, the use of engineered EVs for immunomodulation represents a cutting-edge research direction that combines recent advances in nanotechnology and molecular biology [140]. As our understanding of EV biology and immunomodulatory mechanisms continues to evolve, it is possible that these types of technologies will be employed in clinical therapies in the future [141].

### 4.7. MicroRNAs

MicroRNAs are important for regulating gene expression [142]. Studies have shown differences in microRNA expression between tumor and normal cells, which may affect macrophage polarization [53]. On the one hand, some microRNAs that are highly expressed in tumor cells tend to regulate the differentiation of macrophages to M2 macrophages, creating better survival conditions for cancer cells. [143,144,145]. For instance, studies have demonstrated that cancer cells with mutant p53 can alter the function of macrophages to support tumor growth by utilizing miR-1246 [146]. Similarly, another group of microRNAs regulates macrophage differentiation into M1 macrophages, which enhances their ability to kill tumor cells [147]. Wang et al. found that miR-125a could reverse the TAM phenotype, giving it an antitumor effect [148]. Yang et al. conducted a study on the correlation between miR-506 and macrophages within the tumor microenvironment. Their findings revealed that miR-506 could reprogram M2-like macrophages into an M1-like phenotype by targeting STAT3, thus offering novel insights and potential strategies for macrophage reprogramming in this context [131].

### 4.8. Synergistic Effects

All the above methods can reprogram M2 to M1 macrophages. However, the anti-cancer effect of using a single method alone is limited [149]. People gradually turn to synergistic therapy [150]. Compared with monotherapy, synergistic therapy can produce more effective cancer cell inhibition. For instance, the combination of engineered EVs with photodynamic therapy proved to be more effective in treating colorectal cancer than monotherapy [136]. The combination of different drugs may produce unexpected results. For instance, Liao et al. used iron oxide nanoparticles in combination with lactate oxidase to treat M2 macrophages and differentiate them from M1 macrophages. This method may potentially augment immune checkpoint inhibitor therapy for cancer [18]. Synergistic therapy may be highly effective in immunotherapy, especially in overcoming the limitations of monotherapy [151]. As our understanding of the interaction between the immune system and the tumor microenvironment continues to improve, effective synergistic therapeutic strategies targeting M2 to M1 macrophage transformation may be developed in the future [152].

## 5. Conclusions and Future Perspectives

More and more evidence suggests that extracellular vesicles and extracellular vesicle microRNAs secreted by M1 macrophages play a significant role in cancer treatment [54,57,59,60]. These molecules play a significant role in inhibiting the development and metastasis of various cancers, including glioma [57], lung cancer [59], hepatocellular carcinoma [60] and gastric cancer [54]. EV microRNAs can inhibit cancer cell proliferation, migration, and invasion through intercellular communication [153]. Furthermore, EVs secreted by M1 macrophages are considered one of the best vehicles for drug delivery due to their unique advantages and antitumor effects [75]. M1 macrophages exhibit bactericidal and antitumor effects, while M2 macrophages promote an immunosuppressive response and facilitate cancer progression [154]. The transformation of M2 macrophages into M1 macrophages is crucial for potential cancer therapies [155]. Currently, pharmacologists have developed several drugs related to this process. In conclusion, M1 macrophages and their EVs are expected to play an important role in cancer therapy and may serve as novel tools for cancer diagnosis and treatment [156]. It is worth noting that this is still an ongoing research field, and further research is needed to fully understand its potential.

Many basic and clinical studies have indicated that TAM is positively correlated with tumor development and metastasis [157]. TAMs have the potential to become promising target cells in tumor therapy. However, their heterogeneity poses challenges in studying tumor mechanisms and developing drugs that target TAMs [158]. Analyzing the heterogeneity of TAMs can aid in understanding the mechanisms of tumor progression and drug resistance and may provide potential therapeutic strategies for cancer patients. Single-cell RNA sequencing (scRNA-seq) can be used to reveal the RNA expression profile of each TAM and distinguish their heterogeneity [159]. This provides more effective detection methods and accurate information for TAM-related research. Additionally, scRNA-seq can identify new macrophage subpopulations, map the TME at the single-cell level, identify potential prognostic markers, and analyze interactions between TAMs and other cells in the TME [160]. The current application areas of scRNA-seq in TAM heterogeneity are focused on glioma, breast cancer, and lung cancer [53,161,162]. By combining multiple histological techniques, we believe that more cellular and molecular targets of TAMs can be explored. This could promote TAMs as targets and drug-delivery vehicles for cancer immunotherapy. 

## Figures and Tables

**Table 1:** A summary of the various cargoes delivered by M1-EVs for different oncology treatments.

Cancer Types	Delivery Drug Types
Pancreatic cancer	GEM+ DFX [74]
Breast cancer	PTX [77], DTX [69]
Ovarian cancer	Cisplatin [70,78]
Lung cancer	Cisplatin [76], microRNA-let-7b-5p-GNG5 axis [55]
Bladder cancer	GEM [75]

**Table 2:** The methods and principles of reprogramming M2 macrophages into M1 macrophages.

Methods	Principles	References
Antioxidants	Reducing levels of extracellular oxidation capable of promoting M2 macrophage production using antioxidants.	[90,91,92,93,94,95,96,97,98,99,100]
Photodynamic synergistic therapy	The extracellular ROS generated by type I photosensitizers plays a role.	[101,102,103,104,105,106]
Epigenetic therapy	Regulation of chromatin structure and gene expression using epigenetic approaches.	[107,108,109,110,111,112,113,114]
Natural materials and synthetic drugs	Targeting molecules and signaling pathways that play crucial roles in macrophage polarization.	[115,116,117,118,119,120,121,122,123,124,125,126,127,128,129,130,131,132,133,134,135,136]
Engineering EVs	EVs are modified and encapsulated with drugs and then targeted to M2 macrophages.	[137,138,139,140,141]
MicroRNAs	MicroRNA affects the polarization state of macrophages by regulating gene expression.	[53,142,143,144,145,146,147,148]
Synergistic effects	Multiple methods are combined to achieve reprogramming of M2 macrophages for better anti-cancer effects.	[149,150,151,152]

**Table 3:** Macrophage reprogramming molecules and targets.

Functional Molecules	Targets
6-Gingerol [117]	Arginase
Paclitaxel [118]	TLR4
ZA [119]	TLR4
CHA [121]	JAK-STAT1 and NF-κB pathways
CD40 agonists [123]	CD40 receptor
HRG [124]	Toll-like receptor
Nocardia rubra cell wall skeleton [128]	STAT1/STAT6 pathways
TLR4 Agonist [129]	TLR4
MnTE-2-PyP [130]	Stat6
MiR-506 [131]	STAT3
Lachnum polysaccharide [132]	TLR4
M1 EVs engineered to carry NF-κB p50 siRNA and miR-511–3p [133]	IL4R
Lipo-MP-LPS [129]	TLR4
OX40L M1-exos [134]	OX40/OX40L pathway
5-aza-dC and TSA [112]	miR-7083-5p
LPFe3O4 NPs [135]	NO
IL4R-Exo [133]	IL4R
M1-EVs nanoprobe [136]	ROS, NO
The iron oxide nanoparticles combined with LOX [18]	Carboxylic acid

## Data Availability

Data sharing is not applicable to this study because no datasets were generated or analyzed during the current study.
